# fMRI Under Sedation: What Is the Best Choice in Children?

**DOI:** 10.4021/jocmr1047w

**Published:** 2012-11-11

**Authors:** Byron Bernal, Sandra Grossman, Rafael Gonzalez, Nolan Altman

**Affiliations:** a Department of Radiology, Miami Children’s Hospital, USA; b Department of Anesthesia, Miami Children’s Hospital, USA

**Keywords:** Epilepsy, Auditory mapping, Visual mapping, “Functional MRI”

## Abstract

**Background:**

Pediatric fMRI may require sedation. The aim of this study is to compare different sedation schemes to determine which medication yields least failures and the best activation.

**Methods:**

A total of 100 children who had fMRI performed as part of the work up for epilepsy surgery, were divided into different medication groups (Pentobarbital, Propofol, Dexmedetomidine, Sevoflurane). Comparison was performed among the groups for number of failures, rank of activation, adverse effects, anesthesia time, and recovery time. The study was approved by the IRB and followed all HIPAA guidelines. BOLD sequences were utilized to perform two block-design paradigms (auditory and visual). The activation was ranked into 5 categories according to the presence and localization of the activation. Descriptive and parametric statistics (ANOVA) were utilized to look for significant differences.

**Results:**

Pentobarbital yielded the least amount of failures, for the auditory task, followed by propofol, while sevoflurane yielded the highest number of failures for both tasks. In the visual task, propofol administered after dexmedetomidine resulted in the least number of failures. Brain activations were not statistical different (auditory: ANOVA, P = 0.42; F = 1.01; visual: ANOVA, P = 0.077; F = 2.1). The shortest recovery time was obtained with sevoflurane, followed by propofol. Agitation and cardiac complications were seen in 28% of cases in the pentobarbital group.

**Conclusion:**

No statistically significant difference in brain activation was found utilizing different sedative medications in children with intractable epilepsy. A trend toward less failures was obtained with pentobarbital and propofol; however pentobarbital was more frequently associated with undesirable side effects.

## Introduction

fMRI, although mainly utilized in awake adult patients, is currently being done on pediatric patients of all ages, most of whom are uncooperative. Several articles have reported consistent brain activation for auditory and visual functions in children less than 5 years of age who had fMRI done under sedation. These studies describe a number of different sedatives and anesthetics, oftentimes used in combination depending on prior experience and preference of each specialized center. However, there is a paucity of research regarding the best/most effective sedative scheme for fMRI in the pediatric population.

Theoretically, the sedative of choice should be one that is safe, with a short induction time, few collateral effects, quick recovery and without or very limited effects on brain vascular reaction and neuronal metabolism and response. The aim of this study was to compare different sedation schemes in a clinical pediatric radiology facility, and determine which medication resulted in the best auditory and visual activation in task-related-fMRI paradigms.

## Materials and Methods

This was a retrospective chart review/data analysis of patients who were referred to the Department of Radiology at Miami Children’s Hospital for a functional MRI as part of the work-up for epilepsy surgery, between January 2006 and January 2009.

### Subjects

A total of 105 charts were reviewed after approval was granted by the Western Institutional Review Board (WIRB). Consent was not required, as per the WIRB. All HIPAA regulation policies were observed. Five patients’ data were discarded: 2 due to data corruption; 2 for unavailability of clinical information; and 1 case considered to be an outlier for the combination of sedative medications received. The remaining 100 subjects were divided into 6 different medication groups: Pentobarbital alone (Pen); Pentobarbital with any other medication, usually used as induction (Pen+); Propofol (Pro); Dexmedetomidine (Precedex^TM^) (designated as Dex); Dexmedetomidine-propofol (Dex-Pro); and Sevoflurane (Sev). Each sedation scheme was utilized during a specific and arbitrary decided period of time; thus, patients are consecutive within the same group.

The Dex-Pro group initially received dexmedetomidine alone, and the auditory task was given under this medication. Once the auditory task was completed (task duration: 4 min), the sedation was shifted to Propofol and the visual paradigm was given. This scheme was tried based on previous reports [1] and our own empirical observations. There were no major differences in pathology among the groups, mostly consisting of patients with cortical development anormalities and non-lesional cases. No cases with overt occipital or primary auditory areas cortical dysplasias were included in the study.

### Sedation procedures

All patients were sedated and monitored in the Department of Radiology by a Board certified pediatric anesthesiologist. The “Pen” group received a bolus administration of the medication once they where in the magnet. The remainder of the groups received a brief induction with sevoflurane or nitrous oxide before the infusion of the sedative that was ultimately given for the procedure. Based on our prior experience endotracheal intubation was avoided and the exam was conducted with the patient breathing room air.

In all cases, vital signs, sedative dose, adverse effects, sedation time, and recovery time was recorded and documented. Sedation time was defined as the time difference between the commencement and end of anesthesia. Recovery time was defined as the time between the “end of procedure” on the anesthesia log and the moment the patient was able to drink water, as documented on the nursing notes.

### fMRI procedures

BOLD sensitive echo-planar gradient sequences were utilized in a 1.5 T magnet with an 8-channel SENSE head coil. The fMRI sequences were determined at the start of each session, and consisted of two block-design paradigms. In all cases the auditory task was given first (as per the internal protocol of the Department of Radiology at Miami Children’s Hospital), and consisted of the binaural presentation of either a pre-recorded human voice narrating a story (for children over 1 year of age and normal language development), or a pre-recorded speech by his/her mother. The control condition consisted of no stimulus.

The visual paradigm consisted of flashing lights displayed through the closed eyes, using special goggles. Both paradigms consisted of eleven 20-second-epochs -5 on, 6 off, with 10 timepoints per epoch, for a total of 110 timepoints per run. The common settings were: TR/TE/FA = 2,000 ms, 45 ms, 90 ms. Axial cuts of 4.5 mm with no gap, taken in an inferior to superior interleaved manner. The FOV was 240 and the matrix 64 × 64.

### fMRI post-processing

The data was spatially corrected for motion, and smoothed using a Gaussian filter at full-width and half-maximum (FWHM) of 7 mm. Global intensity normalization and linear detrending were applied. Activations were obtained with FSL (FSL library: www.fmrib.ox.ac.uk/fsl), utilizing a general linear model with local autocorrelation correction [2] and a cluster thresholding technique determined by Z > 2.3 and a (corrected) cluster significance threshold of P = 0.05. Activations and de-activations were obtained for the visual paradigm. Areas of activation were co-registered and overlaid into the 2D and 3D anatomical images of the patient.

Activations were evaluated in each case by one of the investigators (BB) with 10 years of experience in the field of pediatric fMRI. The activation was ranked into 5 categories accordingly with the following criteria: 0 (none) = no activation shown; 1 (poor) = no activation in canonical areas but some outside of them; 2 (fair) = weak, scattered activation in canonical areas with or without some noise in other brain areas; 3 (good) = small well defined areas of activation in canonical areas with no or minimal activation elsewhere; 4 (excellent) = well defined areas of activation in canonical areas with no or minimal activation elsewhere.

**Figure 1:**
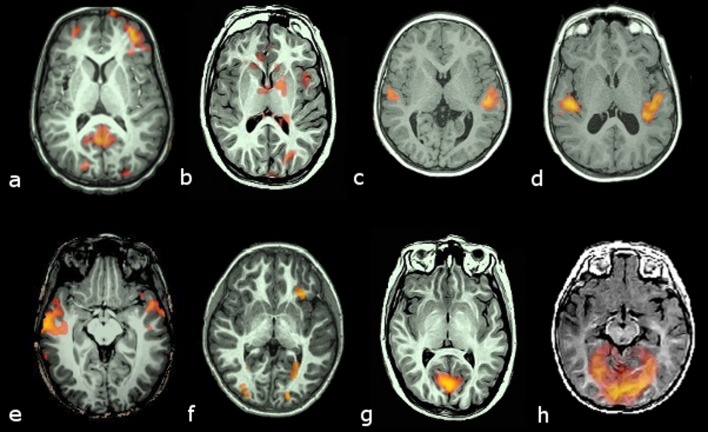
Examples of activation ranking. Transversal slices in radiological convention (left hemisphere on the right side) of 8 cases with different brain activation types. Activation maps are color coded from bright yellow (highest) to red (lowest) intensities. Upper row (a to d) corresponds to activations obtained with the auditory stimulus. Lower row (e to h) corresponds to activations obtained with the visual stimulus: Rank 1, no activation in canonical areas (planum temporale for the auditory or medial occipital lobe for the visual), but some activation outside them (a, e); Rank 2, weak and scattered activation in the canonical areas, with some activation in other areas (b, f); Rank 3, small but otherwise well defined areas of activation of canonical areas (c, g); Rank 4, well defined and large activation of target areas (d, h).

Ranks of 0 and 1 were deemed as failure of activation. Examples of activations are given in Figure 1.

### Data analysis

The number of fMRI failures was annotated for each group. Within each group, the cases with activation were pooled to find the mean and the variability of the activation, and these results were compared. “Time of sedation” and “time of recovery” were also compared amongst the groups. Complications from the sedation were documented. Descriptive statistics were used for the failures. Parametric statistics (ANOVA) were utilized to look for significant differences in the sedative-subgroups, via the software PSPP (http://www.gnu.org/software/pspp/). Post-hoc analysis (two tailed t-test) was implemented in order to compare differences in activation between the most relevant candidates. The null hypothesis (no differences between the means) was rejected for p values < 0.05, and Bonferroni corrected, for multiple comparisons.

## Results

No statistical significant differences of age was found among the groups (ANOVA: P = 0.75; F = 0.48). All subjects completed their functional and structural studies with the sedation chosen for them. Table 1 summarizes the demographics of the patients according to the medication that was given for sedation.

**Table 1:** Patient Demographics According to Sedation Medication Administered

Item	Pen	Pen+	Pro	Dex	Dex-Pro	Sev	Total
N	17	13	19	24	18	9	100
Male	11	8	9	14	13	4	59
Female	6	5	10	10	5	5	41
Age mean	5.3	5.7	6.16	6.0	7.1	6.9	6.23
Age range	1 - 13	9m - 14	1 - 14	8m - 14	1 - 17	1 - 19	1 - 19
Mean dose	4.64 (*)	3.26	97.9 (**)	1.8 (***)	0.7/90	0.5 MAC	
Dose range	3.0 - 8.0	2.5 - 5.0	50 - 100	0.8 (***)	0.5 - 0.9/50 - 200	0.3 - 0.75 MAC	

*: mg/kg; **: μg/kg/m; ***: μg/kg/hr. Pen was administered by bolus; Pro and Dex were administered by infusion.

Tables 2, 3, 4, 5, and 6 summarize the findings. In the auditory task, pentobarbital administered alone was the medication with the least amount of failures, followed by propofol. Sevoflurane yielded more failures. In the visual task, propofol administered after dexmedetomidine resulted in the least amount of failures, followed by pentobarbital alone. Dexmedetomidine given alone caused the highest number of failures in the visual task.

**Table 2:** Failure Rates (No Activation Obtained in Canonical (Auditory or Visual) Areas

Medication	Auditory Task	Visual Task
Pen	18% (3/17)	25% (4/16)
Pen +	38% (5/13)	15% (2/13)
Pro	21% (4/19)	39% (7/18)
Dex	25% (6/24)	46% (11/24)
Dex-Pro	28% (5/18)	11% (2/18)
Sev	44% (4/9)	44% (4/9)
Average	0.29	0.315

**Table 3:** Activation Intensity by Medication (Including Ranks 0 and 1)

	Number	Auditory mean	Auditory SD	Visual mean	Visual SD	
Pen	17/16(*)	3.0	1.37	2.81	1.52	
Pen+	13	2.31	1.7	3.00	1.22	
Pro	19/18	2.37	1.38	2.28	1.78	
Dex	24	2.54	1.64	2.04	1.9	
Dex-Pro	18	2.83	1.54	3.17	1.25	
Sev	9	1.78	1.39	1.56	1.51	
ANOVA Ho =						(Auditory) P = 0.42
						(Visual) P = 0.077

Ranking means includes all ranks (0 to 4). (*) = Auditory and visual, respectively.

**Table 4:** Activation Intensity of Cases With Activation in Canonical Areas (Rank 2 to 4)

	Auditory - mean	Auditory - SD	Visual - mean	Visual - SD	
Pen	3.5	0.85	3.58	0.67	
Pen+	3.5	0.76	3.45	0.52	
Pro	2.93	0.88	3.55	0.82	
Dex	3.39	0.78	3.69	0.63	
Dex-Pro	3.69	0.63	3.56	0.51	
Sev	2.8	0.84	2.8	0.45	
ANOVA					(A) P = 0.1
Ho =					(V) P = 0.12

The null hypothesis was not rejected for either group (Auditory: ANOVA P = 0.1; F = 1.95); (Visual: P = 0.17, F = 1.61).

**Table 5:** Duration of Sedation

Medication	Time - mean (minutes)	Time - SD	
Pen	98.8	31.10	
Pen+	144.27	17.85	
Pro	126.58	37.04	
Dex	148.83	28.87	
Dex-Pro	151.44	13.56	
Sev	140.56	22.84	
ANOVA			P = 0.0005
			F = 7.85

**Table 6:** Recovery Time

Medication	Time - mean (minutes)	Time - SD	
Pen	47.0	35.67	
Pen+	27.93	14.9	
Pro	18.47	14.35	
Dex	28.92	15.15	
Dex-Pro	28.11	15.95	
Sev	17.33	4.39	
ANOVA			P = 0.0005
			F = 4.47

Ranking the activations per group we obtained the results shown in Table 3.

In this analysis (ANOVA), there were no statistical differences among the group means for either task (Auditory: ANOVA, P = 0.42; F = 1.01); (Visual: ANOVA, P = 0.077; F = 2.1).

We further analyzed the data to compare all groups subjects with canonical activation, that is, patients with activation ranking between 2 and 4 (Table 4). In this analysis, the scheme with the most robust auditory activation was pentobarbital alone, followed by dexmedetomidine (when preceding propofol). The worst activation was seen with sevoflurane. The best visual activation was obtained with pentobarbital alone, followed by propofol when administered just after dexmedetomidine.

### Sedation time and recovery time

Table 5 shows the sedation time per group.

ANOVA analysis of the distribution of means strongly rejects the null hypothesis (P = 0.0005; F = 7.85), even if the group is recalculated without Pen (P = 0.005). The post-hoc comparison of Pen vs Pro (closest in rank) shows a significance of P = 0.021. However, this difference reflects the fact that Pen was used longer ago than the rest of the medications. By then, the MRI protocol included less sequences and the patient was sedated directly in the scanner room. More importantly, there was a statistically significant difference between Pro vs Dex (P = 0.0324).

ANOVA analysis of the recovery time also showed a significant differences (P = 0.0005) (Table 6).

The medication with the shortest recovery time was sevoflurane, followed by propofol. Post-hoc analysis using propofol as the reference showed significant statistical differences with Pen (paired t-test, P = 0.003), Dex (P = 0.023). The comparison between Pro and Sev did not yield a statistical difference (P = 0.8).

### Sedation complications and recovery side effects

Nine cases showed either intra-session complications or undesirable side effects during recovery (9%, 9/100), 3 cases showed overt agitation during the sedation period, 2 with pentobarbital and 1 with dexmedetomidine. In both cases propofol was added, and thus entered the group of Pen+ and Dex-Pro. One case under Pen developed bradycardia (HR < 56) and another case showed laryngospasm with propofol. Within the recovery period 5 cases showed agitation, 2 from the Pen+ group, 1 from Pen and 2 from Sev group. One case from the Pen group showed atrial flutter, requiring special clinical attention. A total of 7 different cases within both Pen and Pen+ (n = 25) developed either agitation or cardiologic complications (28%).

## Discussion

### Importance of fMRI in pediatrics

Brain mapping is a common practice in the workup for brain surgery, particularly in epilepsy surgery, aimed at sparing well developed areas. Prior to the advent of fMRI brain mapping was exclusively performed with invasive methods, such as direct cortical electrical stimulation performed on an awake subject. Since the inception of fMRI as a technique to map brain functions invasive methods have been limited to very specific cases. However, both intraoperative brain mapping and fMRI (extraoperative) require cooperation of the subject. Therefore, brain mapping is usually not seen as being feasible in small children, particularly with regard to language and memory functions.

Brain activation has been obtained in sedated adults and children with standard fMRI utilizing passive tasks. Auditory and visual activations have been reported by several authors since 1998 [3-6]. Although studies are scarce, the procedure is currently suitable for uncooperative children and mentally handicapped patients.

The availabiltiy of auditory fMRI mapping for uncooperative patients is of great importance. Primary auditory asymmetries that can be demonstrated under sedation might be associated with true language lateralization. This is currently being investigated and several prior findings from different neurophysiology fields seem to validate this association. Several groups have independently found that early (up to two months of age) skills of phonological discrimination are lateralized, and correlated with language lateralization at later ages [7-12]. Hence, fMRI under sedation may be a tool capable of providing clues of language lateralization, thus guiding neurosurgeons towards more or less liberal resections.

Currently, there are a few studies on sedative or anesthetic drugs influence on the BOLD effect. As a result, questions relevant to the sedative of choice, its collateral effects, failure rates, effects in brain activation and vascular response impact (effect in the BOLD response) are still not resolved.

Previous works have described several sedative alternatives, mostly with medications having a rapid onset of action. Propofol [1, 5, 13, 14], pentobarbital [4, 6, 15-18], sevofluorane [19, 20], dexmedetomidine [21], and midazolam [5, 21] are the most frequently mentioned and theoretically the best for this indication. All of these aforementioned drugs have pros and cons. Activation of the auditory cortex has been found to decrease as plasma levels of propofol surpases 0.5 μg/mL [1]. Visual activation decreases when sevofluorane is given below or above 0.75 minimum alveolar concentration (MAC) [22]. Pentobarbital is known to be associated with cardiologic complications.

There are only a few of comparative studies on this subject matter. Some authors report discriminative usage of pentobarbital and chloral hydrate depending on the age of the patient, but do not report the differences between the sedatives, partly because this was not the specific aim of the studies [4, 6, 18]. Gemma and coworkers [5] contrasted brain auditory activation with propofol and midazolam in 14 children between 3 and 7 years of age. The authors found that propofol activation occurring in the primary auditory area was quite similar to normal activation seen in adult awake patients. Activation obtained with midazolam appeared mostly out of the canonical areas and was scattered. Martin et al [15] studied visual brain activation with 3 different anesthetics: halothane, chloral hydrate and pentobarbital. They did not find any differences among the groups for the positive activation, although they did for deactivation. Coull et al [21] compared dexmedetomidine and midazolam in a group of adult volunteers, and demonstrated drug-dependent anatomical variations in activation.

Our study compared different sedation alternatives. Although no statistically significant findings were obtained to decisively choose one option as the best one, our study nonetheless yielded important findings. Pentobarbital was the medication resulting in the least amount of failures in auditory mapping, although it was also the medication showing more side effects and complications. Obtaining auditory activation may be crucial since, in our experience, it lateralizes to speech perception early in life, and such lateralization may be eventually correlated to language localization [12, 23]. Sevoflurane yielded too many failures and seems to be an anesthetic to avoid, a finding that seems to be supported by previous publications [19, 20, 24]. When visual activation is the main target, pentobarbital, or propofol alone or after induction with dexmedetomidine, are felt to be the best options since they yielded less failures, not because they produced higher activation.

Sedation times are also of practical interest. Our study showed a statistically significant difference in this area. The short sedation times associated with pentobarbital reflects two factors. First, the pentobarbital group (Pen) consisted of cases performed earlier during the study. At that time, our brain protocol for epilepsy patients consisted of fewer sequences. In addition, the induction with pentobarbital was done in the scanner room and not in a dedicated induction room as in the rest of the cases. Excluding (Pen) it was interesting to note that there was a statistically significant difference between propofol and dexmedetomedine. Therefore, propofol seems to be the sedative of choice when time is the main concern. Propofol is also the medication with the shortest recovery time, after sevoflurane.

The choice of the best sedation drug for fMRI not only has to do with how much of the BOLD effect is preserved, but also the extent of undesirable and harmful side effects. Pentobarbital (alone or in combination) yielded the highest number of collateral effects, ranging from agitation to atrial flutter. 28% of the subjects receiving this sedative had undesirable side effects or complications, which is a deterrent to using pentobarbital particularly in non-hospital outpatient settings.

Our study had several limitations that were mostly due to its retrospective design. The authors adopted several strategies to help minimize the impact of the inhomogeneity resulting from the lack of a pre-defined protocol (as would be expected in a retrospective study). First, subjects entering the study were seen by the same medical group (except for the pentobarbital group). This medical group had an internal standard protocol for monitoring patients’ vital signs and providing annotated information on dose, start and end time. Likewise, our nurses followed standard guidelines when providing information in their written notes regarding the recovery of the patient. Whenever the authors found incomplete information/documentation, that case was excluded from the study, however, this occurred in very few cases.

Also, we did not have drug titration with plasma levels. However, the doses that were given in all cases were in accordance with prior established guidelines and our own experience with keeping doses low enough to find brain activation but high enough to remain motionless maintaining stable hemodynamic and respiratory parameters. We feel that the doses were comparative. When isolated cases were medicated with lower doses, the patient awoke.

A single rater review of the data may be an important limitation. One of the authors (NA) was not involved as a rater since he was directly involved in the clinical reporting of the fMR exams. The possibility of associating the fMR activation patterns with known case-specific outcomes could bias the grading. Similar expertise in fMRI is limited, therefore, the authors decided that adding an external reviewer without similar expertise would degrade the rating method unnecessarily.

Further research is needed with controlled studies and significantly more subjects in order to reach more precise conclusions. The interaction between combined medications and brain activation is a field ripe for future research, as illustrated in our work by the striking results seen when giving dexmedetomedine for auditory and propofol for visual mapping. The effect of this sequential combination is not well understood. Nonetheless, our study could be used as a guideline in clinical settings.

### Conclusions

We present a retrospective study of auditory and visual fMRI mapping in 100 patients under sedation with different pharmaceutical agents. We found no statistically significant differences but several trends were noted. Less failures were obtained with pentobarbital and propofol, but pentobarbital was more frequently associated with undesirable side effects and cardiologic complications. More studies are needed to help clarify the effects of sedation schemes combining various medications on fMRI auditory and visual activation as we found this method successful in our experience.
